# Assessing low-back loading during lifting using personalized electromyography-driven trunk models and NIOSH-based risk levels

**DOI:** 10.3389/fbioe.2025.1486931

**Published:** 2025-02-07

**Authors:** Mohamed Irfan Refai, Tiwana Varrecchia, Giorgia Chini, Alberto Ranavolo, Massimo Sartori

**Affiliations:** ^1^ Department of Biomechanical Engineering, University of Twente, Enschede, Netherlands; ^2^ Department of Occupational and Environmental Medicine, Epidemiology and Hygiene, National Institute for Insurance Against Accidents at Work, Rome, Italy

**Keywords:** workplace musculoskeletal disorder, NIOSH, musculoskeletal modelling, lifting, electromyography

## Abstract

Workplace injury risk due to physically demanding tasks (e.g., repeated lifting) is currently assessed using ergonomic guidelines. The Revised NIOSH Lifting Equation (RNLE) is a commonly used approach that assesses risk of low-back loading during different lifting tasks. Advances in musculoskeletal models have enabled the estimation of physiologically valid person-specific musculoskeletal models (pEMS) driven by surface electromyography and joint angle information. These models offer realistic estimates of objective parameters such as moments and compressive and shear loads at the lumbosacral joint. In this study, we applied both techniques (RNLE and pEMS) to assess risk and low-back loading in seven healthy participants performing lifting tasks at different risk levels. We found that the pEMS estimated objective parameters of low-back loading in line with the different risk levels proposed by RNLE. However, the low-back compressive and shear loads were higher than the limits proposed by the RNLE. Moreover, we show that the lumbosacral compressive loads can be a better parameter to demarcate risk levels. We recommend performing this assessment on a larger and diverse population for evaluation of personalized risk levels across lifting tasks in the industry. These approaches can be implemented with wearable sensorized garments to monitor personalized musculoskeletal health unobtrusively in the workplace providing us a better insight into possibility of individual risk.

## Introduction

Physically demanding tasks such as repeated manual weight-lifting activities are present in the majority of workplaces. These are the primary causes of work-related low-back disorders (WLBDs) due to forces acting on the spine during the execution of the lifting activities (Bakker et al., 2009; Becker, 2001; Chinichian et al., 2021; Dick et al., 2020; Granata and Marras, 2000; Griffith et al., 2012; Hooftman et al., 2010; Okello et al., 2020; Waters et al., 2011). In this context, WLBDs are the most prevalent musculoskeletal conditions and result in high treatment costs (Waters et al., 2011). They account for between 13% and 24% of all workplace illnesses and injuries, 26%–50% of all reported cases of occupational musculoskeletal disorders, 15%–25% of annual sick leave days, and 25% of workers’ yearly compensation costs (Da Costa and Vieira, 2010; INAIL, 2020; Kim et al., 2010; Kuijer et al., 2014).

Thus, it is crucial to accurately and quantitatively identify the risk of WLBD in individuals during occupational lifting activities for efficient ergonomic interventions (Buckle, 2005; Palmer et al., 2012; Waters et al., 1994). There are several risk assessment methods that focus on application rather than personalized measurement of exposure to risk factors (Stirling et al., 2019). Among these approaches, the Revised NIOSH Lifting Equation (RNLE), established by the National Institute for Occupational Safety and Health (NIOSH), is commonly used to evaluate manual lifting (Waters et al., 1993; Waters et al., 1994). The RNLE approach allows calculating the recommended weight limit (RWL) that could be carried, with minimal risk to the human musculoskeletal system. The RNLE quantifies a lifting index (LI) for a given activity. The lifting activities are thus designed such that the LI is equal to or less than 1.0, corresponding to low risk (Spector et al., 2014). LI allows comparison of the lifting demands associated with different lifting tasks and can indicate the percentage of the workforce that is likely to be at risk for developing lifting-related low-back pain (Waters et al., 1993; Waters et al., 1994).

Despite its widespread use, RNLE does not reflect person-specific loads on the lower back (Arjmand et al., 2015; Gallagher and Marras, 2012; Ghezelbash et al., 2020). This is due to two reasons. First, the equation is applicable only in a limited number of tasks, as the RNLE parameters used exclude its application to many manual lifting operations (Dempsey et al., 2001). This includes tasks such as lifting using only one hand, pushing or pulling, carrying, pushing, or dragging using wheelbarrows or shovels, seated or kneeling, working in a small space, working with unstable objects, moving at a high speed, and working in unfavourable conditions (Waters et al., 1994). Secondly, the RNLE is a generalized set of guidelines that are not personalized to the user’s approach to lifting loads (Ajoudani et al., 2020; Ranavolo et al., 2020; Ranavolo et al., 2018a). This affects how the variables are measured and applied across individuals of different dimensions (Dempsey et al., 2001; Flegal et al., 1991; Marras et al., 1999; Ranavolo et al., 2020; Waters et al., 2007a; Waters et al., 2007b). Studies have shown that compressive and shear forces at the lumbosacral joint are an indicator of injury risk during task performance (Brinckmann et al., 1988). However, these forces are often evaluated with approaches that underestimate their real values (Plamondon et al., 1996; Varrecchia et al., 2024). Objective and personalized measures of joint loading can help understand how the impact of the workplace on an individual. This can help provide the individual with personalized ergonomic support if needed, which can prevent injuries over time. This can help maintain the individual’s quality of life and reduce costs in healthcare to treat WLBDs. Thus, objective and personalized approaches to estimate joint loading are needed for person-specific estimations of loading and risk of injury*.*

Studies have employed simple regression models based on muscle activity or trunk posture to estimate spinal compressions, without accounting for the non-linearities due to force-velocity or force-length relations within the muscle (Arjmand et al., 2011; Mientjes et al., 1999). Previous studies proposed the use of optimization techniques to estimate lumbar muscle activations from posture data (Bazrgari et al., 2007; Cholewicki et al., 1995; Kim and Zhang, 2017; Van Dieën and Kingma, 1999; von Arx et al., 2021). However, these approaches distribute joint moments using *a priori* chosen optimality criteria that do not fully capture diverse multi-muscle co-activation patterns and may not be generalizable to biomechanically different tasks, or changes to muscle activation due to fatigue (Dugan and Frontera, 2000). The amount of spine loading could be underestimated if co-activation is not incorporated in biomechanically consistent modelling formulations (Granata and Marras, 2000; Marras and Granata, 1997; Thelen et al., 1995).

An alternative accurate and robust approach would be to utilize surface electromyography (sEMG) measurements to drive forward person-specific musculoskeletal models (Lloyd and Besier, 2003; Moya-Esteban et al., 2022; Sartori et al., 2012). sEMGs capture muscle electrical activity and thereby person-specific patterns of multi-muscle activation that would be otherwise difficult to estimate via optimization. Our work recently enabled the estimation of the musculoskeletal forces exerted by large sets of muscle-tendon units (>230 units) onto lumbar joints during lifting tasks (Moya-Esteban et al., 2022). This approach does not make *a-priori* assumptions regarding how the muscles share the load around a joint, which is an advantage over static optimization strategies (Bazrgari et al., 2007; Cholewicki et al., 1995; Kim and Zhang, 2017; Van Dieën and Kingma, 1999; von Arx et al., 2021). This allows for generalization across tasks. Such an sEMG-driven modelling pipeline was employed to simulate trunk dynamics for the estimation of joint moments and compressive forces at the lumbosacral joint, within the laboratory across different lifting conditions (Moya-Esteban et al., 2022). This approach offers a data-driven approach to estimate biomechanical differences during lifting and compressive forces. Thus, the sEMG-driven approach offers an objective and physiological approach to estimate low-back loading. However, studies have not assessed the agreement of these personalized measures with the ergonomic risk limits set by RNLE.

Therefore, in this study, we compare for the first time, the variation in objective lumbosacral joint loading (compressive and shear forces) estimated by data-driven person-specific musculoskeletal model estimates against the different risk levels proposed by RNLE. This offers potential insights between personalized biomechanical metrics with empirical risk levels. We hypothesized that the low-back loading estimated by the personalized approach should reflect the different risk levels. Additionally, we assessed whether the objective measures could be used to improve the differentiation in the categorical risk levels during lifting tasks.

## Materials and methods

### Participants

Seven male individuals (mean age 31.71 ± 7.06 years, body mass index (BMI) 24.70 ± 1.80 kg/m2), were recruited for the study. The participants had no history of musculoskeletal conditions, surgery on the upper, lower, or trunk, neurological or orthopaedic illnesses, vestibular system problems, visual impairments, or back pain. Before participating in the study, every participant provided their informed consent, which was accepted by the local ethics committee and adhered to the Helsinki Declaration and had local ethics committee “LAZIO 2” approval (N. 0,078,009/2021).

### Measurement setup

**FIGURE 1:**
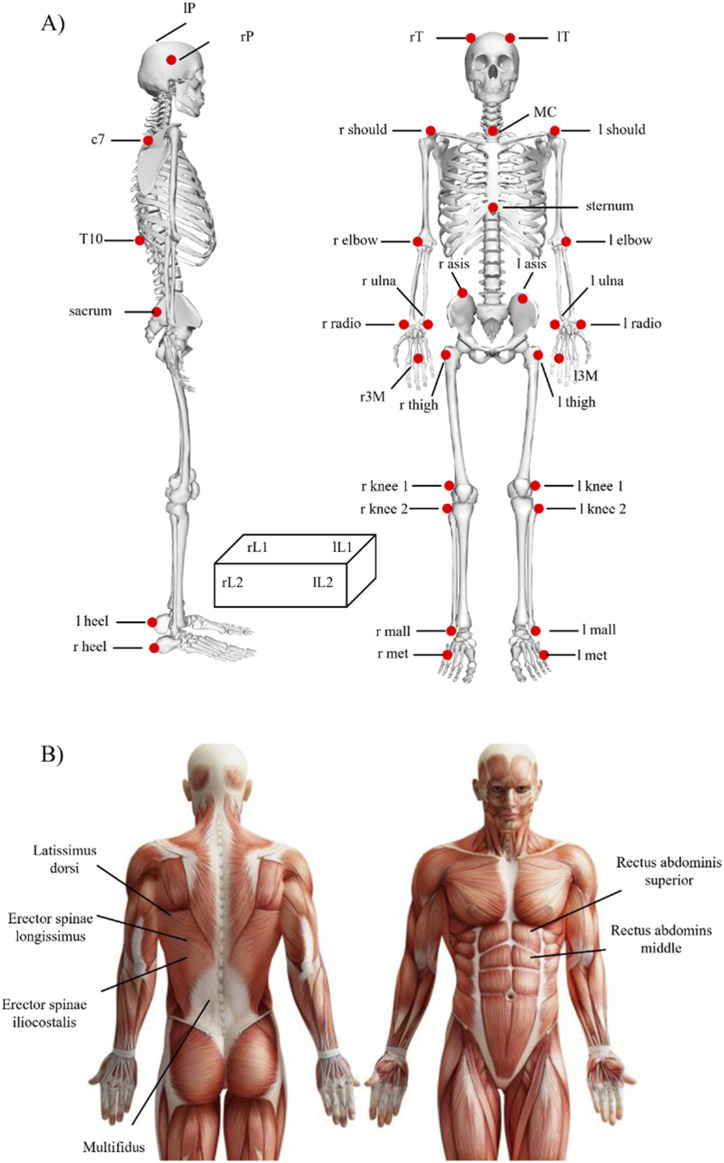
Sensor placement for **(A)** motion capture and **(B)** surface Electromyography signals used in the study.

The three-dimensional trajectories of 33 spherical, retroreflective markers (15 mm in diameter) covered with aluminium powder-based material were detected using an optoelectronic motion analysis system (SMART-DX 6000 System, BTS, Milan, Italy) with eight infrared cameras and a sampling frequency of 340 Hz. The marker placement is shown in Figure 1A. The markers were placed over the cutaneous projections of the spinous processes of the 7th and 10th cervical vertebrae, the suprasternal notch (between the clavicular notches) and, bilaterally, over the acromion, olecranon, ulnar styloid and radial processes, head of the third metacarpal bone, temple, posterior-superior parietal bone, anterior superior iliac spine, great trochanter, lateral femoral condyle, fibula head, lateral malleoli, metatarsal head and heel (Gutierrez-Farewik et al., 2006; Rab et al., 2002; Wu et al., 2002; 2005). Additionally, four markers were set over the four vertices of a load made up of a plastic crate. Four embedded dynamometric platforms (P 6000, BTS, Milan, Italy) sampling at a rate of 680sHz were used to measure the ground response forces.

Using a 16-channel Wi-Fi transmission surface electromyograph, surface Electromyography signals were recorded at a sample rate of 1,000 Hz (FreeEMG300 System, BTS). In accordance with the European Recommendations for surface Electromyography (Hermens et al., 2000) and the Atlas of Muscle Innervation Zones (Barbero et al., 2012), bipolar Ag/AgCl surface electrodes (2-cm diameter; H124SG Kendall ARBO, Tyco Healthcare, Neustadt/Donau, Germany) prepared with electroconductive gel were placed over each muscle (2-cm distance between the centres of the electrodes). Six bipolar electrodes were placed bilaterally on the trunk extensors and flexors as seen in Figure 1B.

Data acquired from the optoelectronic cameras, force platforms and surface electromyography were synchronised. In accordance with the International Society of Biomechanics (Wu et al., 2002; 2005), a global reference system was adopted for the motion capture system. In addition, participants got instruction to become familiar with the assessment processes and perform the lifting activities accurately before measurements began.

Participants performed specific exercises needed to record the isometric maximum voluntary contractions (iMVCs) for each of the muscles investigated (Ranavolo et al., 2018b; Vera-Garcia et al., 2010). To record the iMVC for the trunk extensors (Erector Spinae Longissimus and Iliocostalis), each participant was placed in a prone position, lying on a mattress, and an isometric contraction during a trunk extension against resistance (Vera-Garcia et al., 2010). Each participant was then placed in a supine posture and conducted an isometric contraction during trunk flexion against resistance to get the Rectus Abdominis iMVC (Vera-Garcia et al., 2010).

The 3D marker and force plate data were low pass filtered with cut-off 6 Hz using Butterworth 4th order zero-phase filters for further use.

### Revised NIOSH lifting equation

The RNLE identifies the RWL as the maximum load that healthy workers should be able to lift without running the risk of experiencing associated LBDs (Spector et al., 2014). It is defined as:

RWL=LC×HM×VM×DM×AM×FM×CM (1)

$$\text{LI}=\frac{\text{Load}\;\text{Weight}}{\text{RWL}\;}$$ (2)

where LC is the Load Constant (23 kg), HM is the Horizontal Multiplier, VM is the Vertical Multiplier, DM is the Distance Multiplier, AM is the Asymmetry Multiplier, FM is the Frequency Multiplier, and CM is the Coupling Multiplier (Waters et al., 1993; Waters et al., 1994). These multipliers are derived from task variables that describe the user’s pose during a task. Depending on the definition and variation in the task variables, these multipliers could reduce the allowed RWL. Equation 2 defines the LI as the ratio between the weight of the load being lifted and the task’s RWL.

**FIGURE 2:**
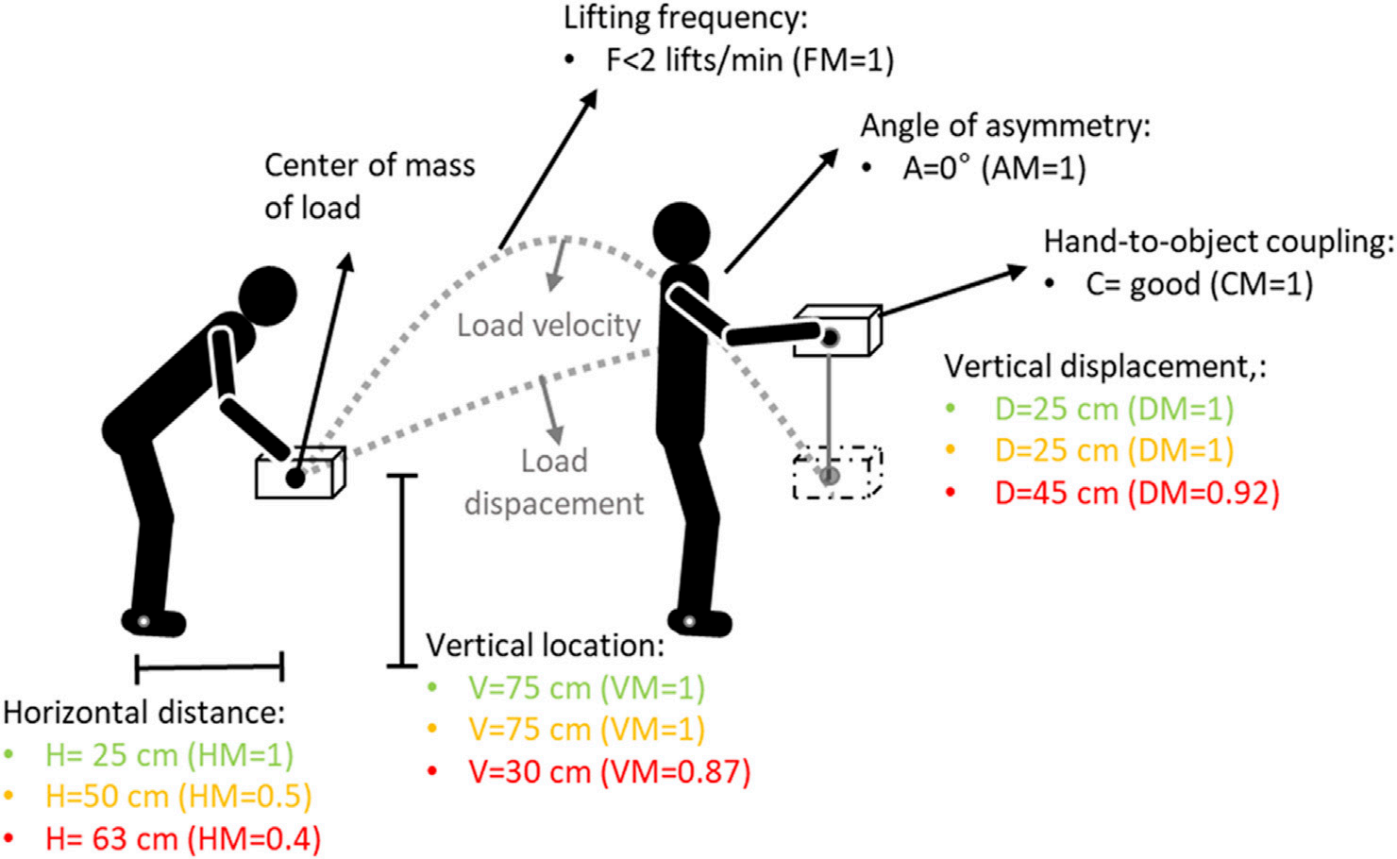
Experimental setup. The lifting conditions for the Lifting Index (LI) equal to 1, 2 and 3 are mentioned in green, yellow and red values respectively. V and VM: vertical locations and corresponding multiplier value; H and HM: horizontal location and corresponding multiplier value; D and DM: vertical travel distance and corresponding multiplier value; F and FM: lifting frequency and corresponding multiplier value; C and CM: hand-to-object coupling and corresponding multiplier value; A and AM: asymmetry angle and corresponding multiplier value. A, F and C for the three risk conditions were equal (Waters et al., 1993; Waters et al., 1994).

In this study, participants performed manual material lifting tasks standing in a neutral body position and lifting a plastic crate with handles using both hands in three different lifting conditions (Figure 2) designed to reflect three different risk conditions with LI values of 1, 2 and 3 (Waters et al., 1993; Waters et al., 1994). To obtain these different LI, the weight, HM, VM, and DM were modified, while the AM, FM and CM were the same for each task. These LI values provide increasing levels of risk comparable to that experienced by the working population (Waters et al., 1993). Tasks with LI values higher than 3.0 are extremely stressful lifting tasks and are associated with a high risk of work-related injuries for a large proportion of workers.

Each participant was required to perform a total of 9 trials (3 repetitions for 3 lifting tasks). The three lifting conditions (LI = 1, 2 and 3) were assigned to each participant in a random order.

### Model-based estimation of lumbosacral joint moments, compressive and shear loads

**FIGURE 3:**
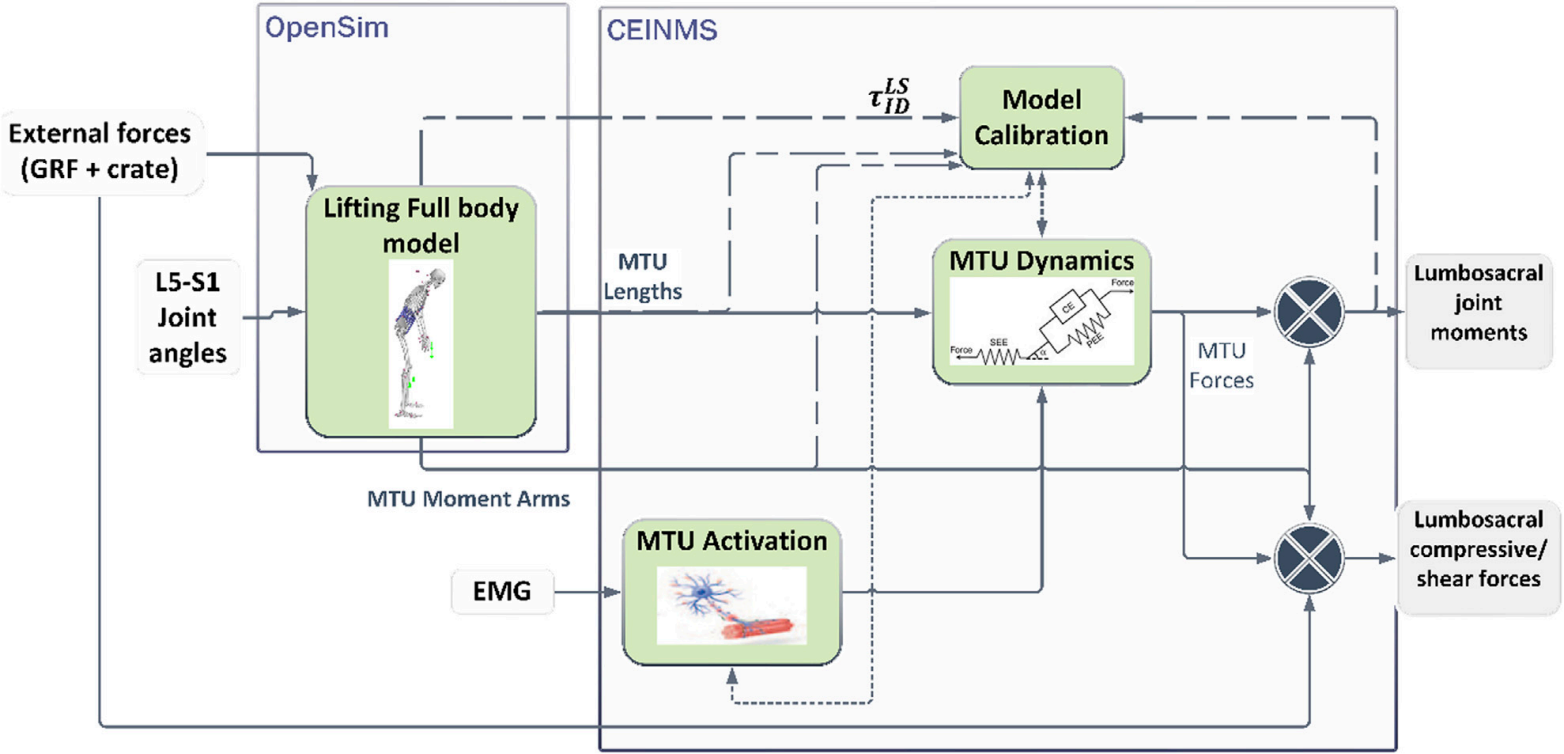
Estimation of lumbosacral joint moments and loads using a person-specific sEMG-driven musculoskeletal model. The Lifting Full Body model developed in OpenSim is used to estimate personalized multi-body dynamics and musculoskeletal geometry (Beaucage-Gauvreau et al., 2019). The CEINMS toolbox was used to personalize the muscle-tendon parameters per participant (Pizzolato et al., 2015) which are denoted by the dashed lines. The dotted lines denote parameters that are used after the calibration to estimate the musculotendon forces during the dynamic tasks.

In this section, we describe the person-specific sEMG-driven musculoskeletal model (pEMS) of the lower back (Figure 3) used for the estimation of joint moments and loads of the lumbosacral joint. For this, first, an OpenSim model was scaled for the participant to obtain multi-body dynamics during the lifting tasks (Beaucage-Gauvreau et al., 2019; Delp et al., 2007). The scaled OpenSim model was then used to personalize the musculotendon parameters using the CEINMS toolbox to obtain the pEMS (Moya-Esteban et al., 2022; Moya-Esteban et al., 2020; Pizzolato et al., 2015; Sartori et al., 2015). Then, we describe how the calibrated model was used to estimate the lumbosacral joint moments, compressive, and shear loads.

### Multi-body dynamics and musculoskeletal geometry

We modelled the plastic crate as boxes with two weights (20 kg and 21 kg) in OpenSim. The 21 kg box was carried by the participant during the LI1 and LI2 tasks, whereas the 20 kg box was carried during the LI3 task. Trajectories of the markers placed on the crate were used for Inverse Kinematics (IK) analyses to get the rotation and movement of the box in 3D. Inverse Dynamics (ID) analyses were then applied to obtain the rotational moments and translational forces exerted by the crate during the lifting tasks.

The Lifting Full Body (LFB) OpenSim model (Beaucage-Gauvreau et al., 2019) designed for symmetric and asymmetric lifting was used for estimating person-specific multi-body dynamics. The LFB model consists of the trunk and 238 Hill-type muscle-tendon units (MTUs) spanning the back and the abdomen. Scaling of the model was done using 3D marker data collected during static movement. Using IK analyses, the lumbosacral joint angle was estimated for each trial using the 3D marker trajectories on the body. All external forces acting on the body, including the ground reaction forces, and translational forces of the crate (applied at the knuckle) were used for the ID analyses to obtain sagittal lumbosacral joint moments. Using the joint angle information, muscle analysis was performed to obtain the MTU lengths and their moment arms around the lumbosacral joint during the lifting tasks.

The IK, ID, and muscle analyses were performed using the OpenSim 4.3 toolbox (Delp et al., 2007).

### Person-specific EMG driven musculoskeletal modeling (pEMS)

The scaled LFB model utilizes generalized MTU parameters such as shape factor of activation dynamics, maximal isometric force, tendon slack length, and optimal fibre length for each MTU present. Therefore, the CEINMS toolbox was used to calibrate these parameters to obtain a personalized pEMS model (Moya-Esteban et al., 2022; Pizzolato et al., 2015). This provides the MTU forces for the specific participant during their lifting tasks.

For calibration of the model, an optimization procedure was performed to identify the optimal MTU parameters by minimizing the difference between the lumbosacral joint moments estimated by the pEMS and the ID analyses from Section 2.4.1. The sEMG envelopes (see Section 2.5) were used to activate the MTU during the lifting tasks (Lloyd and Besier, 2003). Table 1 shows the mapping between the sEMG channels used to record muscle activity and the MTUs modelled in the LFB model. To allow for an optimal model that is suitable for all risk levels, data from the low (LI1) and high (LI3) risk lifting trials were used for calibration.

**TABLE 1:** Mapping the sEMG channels and the respective muscle-tendon units modelled in opensim.

sEMG channels	Modelled muscle-tendon units (MTUs)
Erector spinae longissimus	Longissimus thoracis pars lumborum (10)
	Longissimus thoracis pars thoracis (8)
Erector spinae iliocostalis	Iliocostalis lumborum pars lumborum (42)
	Iliocostalis lumborum pars thoracis (16)
Rectus abdominis middle	Rectus abdominis (2)
	External oblique (12)
	Internal oblique (12)
passive	Quadratus lumborum (36)
	Psoas major (22)
	Multifidus (50)
	Latissimus dorsi (28)

*Note*. The number of MTUs, is represented in brackets.

The calibrated pEMS was then used to estimate MTU forces and lumbosacral joint moments for all trials performed by the participant. The MTU forces and the external forces acting on the body were used to estimate the joint compression and shear forces at the lumbosacral (L5-S1) intervertebral joint using the Joint Reaction Analysis toolbox in OpenSim (Seth et al., 2018). The toolbox estimated the forces acting as a consequence of all loads acting on the model for a given dynamic movement.

### Data processing

First, data for each of the 9 trials was segmented into lift cycles. The lifting cycles were determined by measuring the vertical displacement and velocity of one of the four markers placed over the vertex of the crate, using custom software written in MATLAB (version 2018b, MathWorks, Natick, MA, United States). The start point (when subject starts to lift the weight) was defined when the load velocity exceeded a velocity threshold of 0.025 m/s along the vertical axis while the end of the lifting task was defined where the load velocity fell below the velocity threshold in the opposite direction after the point in which the vertical displacement reached the maximum value (Ranavolo et al., 2015; Ranavolo et al., 2018b; Varrecchia et al., 2018a).

The iMVC and the sEMG raw data were band-pass filtered using a fourth-order Butterworth filter of 20–400 Hz to reduce artefacts and other components of high-frequency noise (Drake and Callaghan, 2006; Ranavolo et al., 2018b). To extract the sEMG envelope a full-wave rectification and low-pass filtering using a fourth-order Butterworth filter at 5 Hz were applied. For each muscle, the sEMG envelope was amplitude-normalized to the average of the three iMVC peak values (Ranavolo 2021; Ranavolo et al., 2018a, Burden, 2010) and time-normalized to the duration of each lifting trial and reduced to 201 samples using a polynomial procedure.

The peak of the normalized sEMG envelopes was evaluated for each participant and trial. We evaluated the range of motion of the lumbosacral joint for the different lifting conditions. This was followed by identifying the peak lumbosacral joint moments in the sagittal plane, and the shear and compressive forces for each trial across the three risk levels of the NIOSH criteria. The joint moments, shear and compressive forces were normalized to the participant’s body weight (BW).

Finally, we assessed if the peak normalized compressive forces offered a higher resolution in risk levels compared to the NIOSH criteria. For this, a cluster analysis using the centroid model was performed on the lumbosacral compressive forces grouped by the lumbosacral moments.

### Data analysis

Statistical analysis was performed to assess the normality of different distributions, and differences between them. First, the distributions of ranges of lumbosacral flexion-extension angles, peak sEMG, peak joint moments, peak shear, and peak compressive forces for each risk level were tested for normality using the Shapiro-Wilk test. The distribution included the parameters from all included trials per participant. The distribution of ranges of lumbosacral flexion-extension angles at all risk levels, peak sEMG for the Rectus Abdominus muscles at all three risk levels, peak moments for the high-risk level, and compressive forces for the medium risk level were not normal (p < 0.05). Therefore, the Wilcoxon ranked sign test was applied with *post hoc* Bonferroni correction to find significant differences between risk levels for each of the biomechanical parameters, and violin plots were used to display the distribution of the values (Bechtold et al., 2021).

The cluster analysis resulted in normally distributed clusters of lumbosacral compressive forces grouped by the lumbosacral moments (p < 0.05). A two-sample *t*-test was applied between the groups of clusters to test if they were significantly different from each other.

All statistical analyses were performed using MATLAB (version 2018b 9.5.0.1178774, MathWorks, Natick, MA, United States).

## Results

**FIGURE 4:**
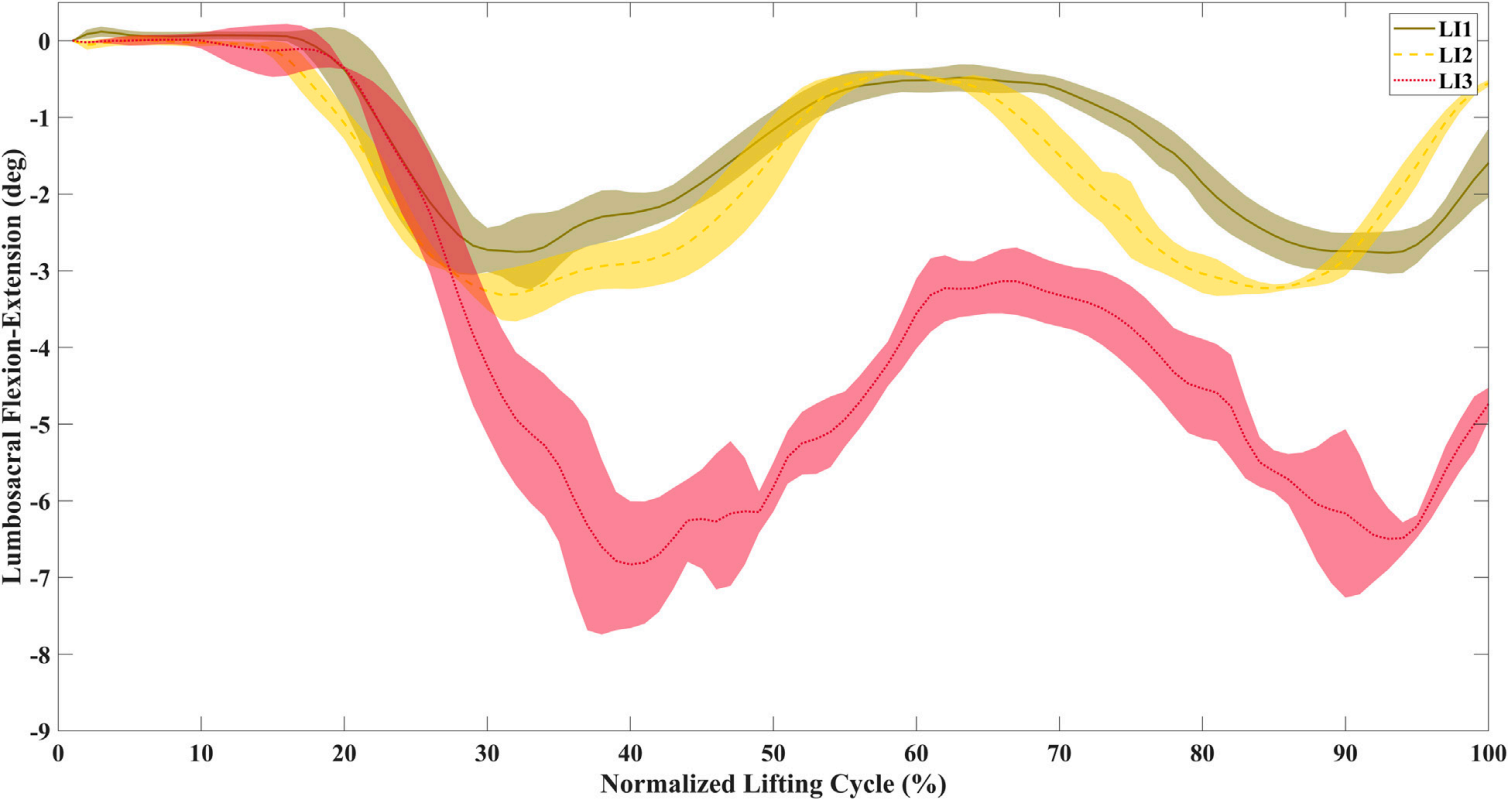
Time-normalized average lumbosacral flexion-extension angles for a representative participant during lifting. The range of motion increases with the increase in risk level.

**FIGURE 5:**
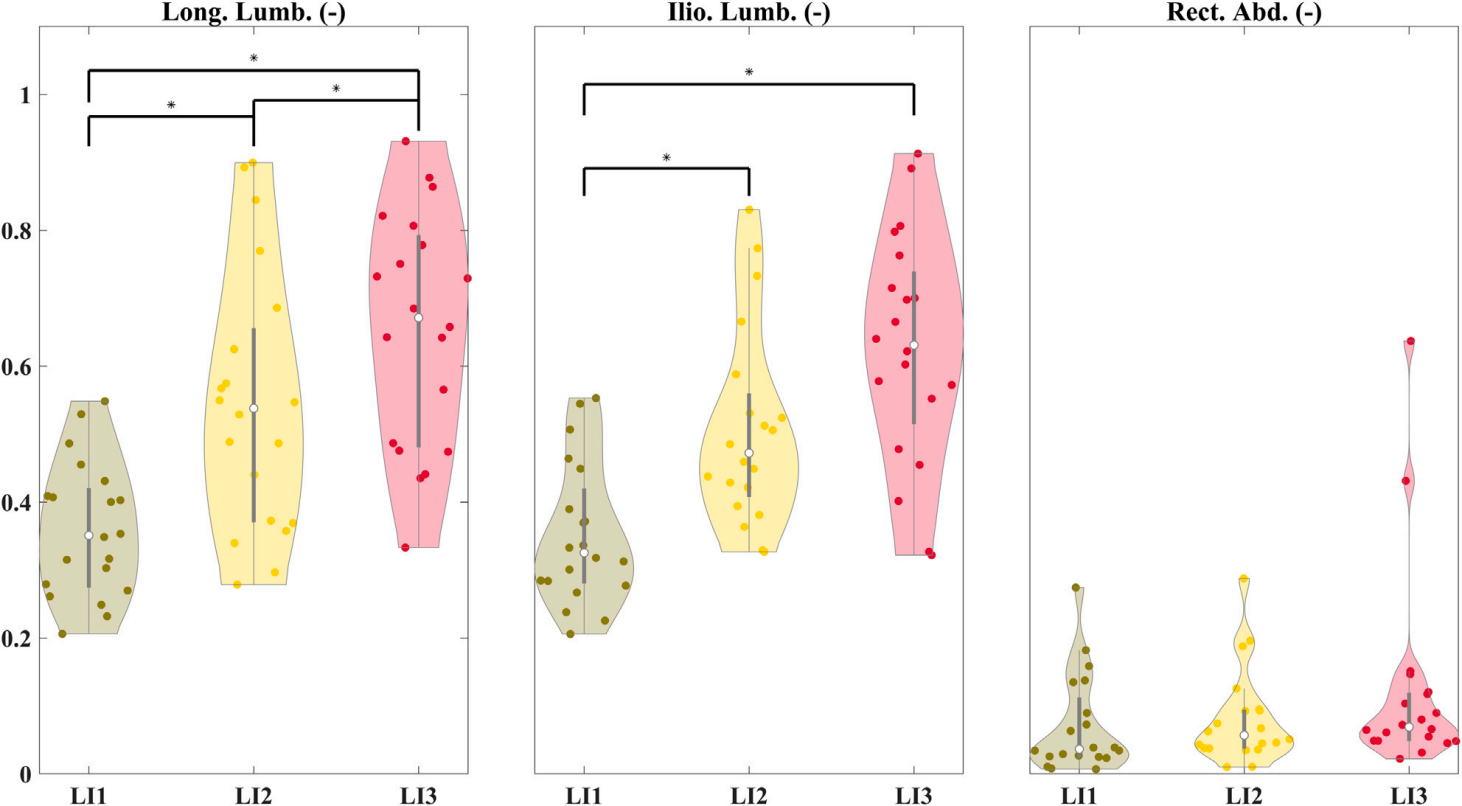
Distribution of peak sEMG averaged between the left and right muscles for the Longissimus Lumborum (Long. Lumb.), Iliocostalis Lumborum (Ilio. Lumb.), and Rectus Abdominus (Rect. Abd.). The peak sEMGs were normalized to the iMVC values. Significant differences are shown between risk levels for the erector spinae and abdominal muscles.

Significant differences were found between the lumbosacral flexion-extension angle ranges for the low and high (z = −4.61, p < 0.01), and medium and high (z = −4.02, p < 0.01) risk levels across participants (Figure 4). Figure 5 shows the distribution of peak sEMG averaged between the left and right muscles. The peak Longissimus Lumborum sEMG was significantly different between the low and medium (z = −3.8, p < 0.01), medium and high (z = −2.58, p < 0.05), and between the low and high-risk levels (z = −3.92, p < 0.01). The Iliocostalis Lumborum activity was also significantly different between the low and medium (z = -3.92, p < 0.01), and low and high (z = −3.73, p < 0.01). The Rectus Abdominus Medialis activity did not show differences between any risk levels.

**FIGURE 6:**
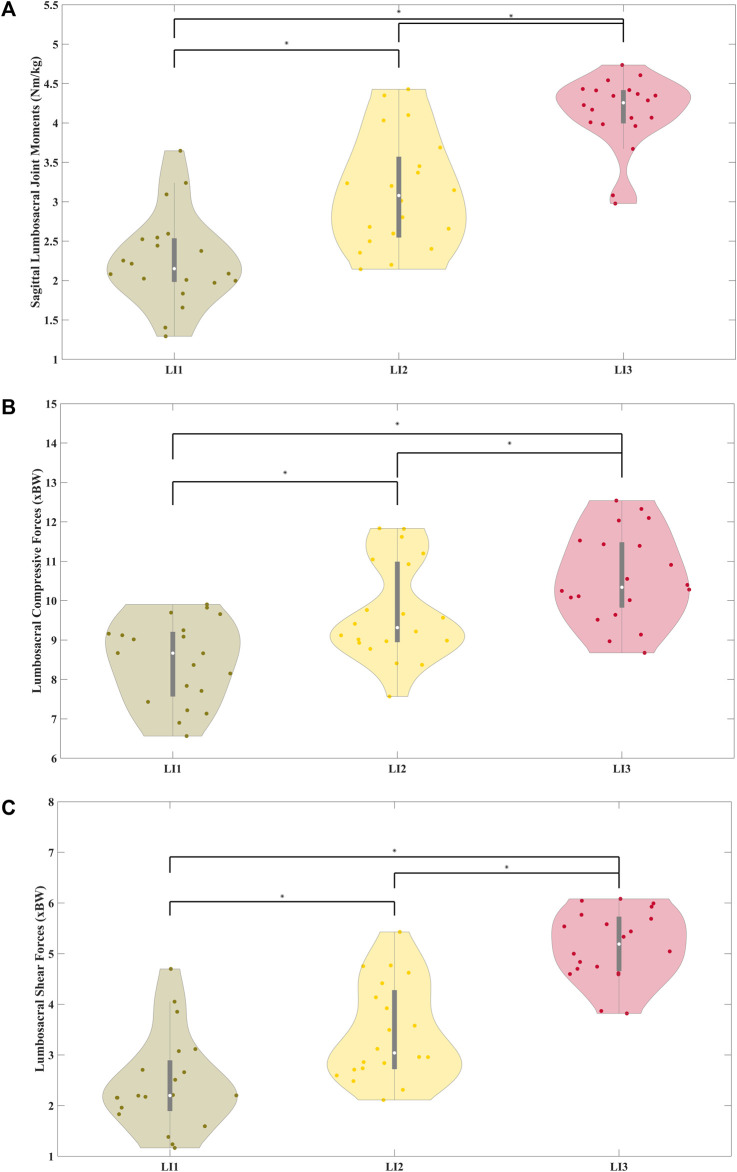
Distribution of body weight normalized peak sagittal lumbosacral joint moments normalized to participant body weight **(A)** and distribution of body of body weight normalized peak compressive **(B)** and shear **(C)** forces averaged across participants for the three risk levels. The joint moments, compressive and shear forces were significantly different across the risk levels.

Figure 6A shows the distribution of peak BW-normalized sagittal lumbosacral moments which were significantly different between levels: low and medium (z = −3.88, p < 0.01), medium and high (z = −2.99, p < 0.01), low and high (z = −3.88, p < 0.01). The average peak moments were estimated to be 2.26 ± 0.58, 3.11 ± 0.71, and 4.14 ± 0.45 Nm/kg for the low, medium, and high-risk levels respectively. The distributions of the peak BW-normalized compressive forces were also significantly different across all three risk levels (p < 0.01) (Figure 6B) (z = −3.8, z = −3.09, and z = −3.84 between the low and medium, medium and high, and low and high-risk levels respectively) (Figure 6B). The average peak compressive forces were estimated to be 8.46 ± 1.02, 9.77 ± 1.18, and 10.74 ± 1.02 times BW for the low, medium, and high-risk levels respectively, whereas the shear forces were 2.49 ± 0.89, 3.48 ± 0.92, and 5.28 ± 0.79 times BW for the low, medium, and high-risk levels respectively. Significant differences (p < 0.01) in peak BW-normalized shear loads were seen between the low and medium (z = −3.88), medium and high (z = −3.55), and low and high (z = −3.88) risk levels (Figure 6C).

**FIGURE 7:**
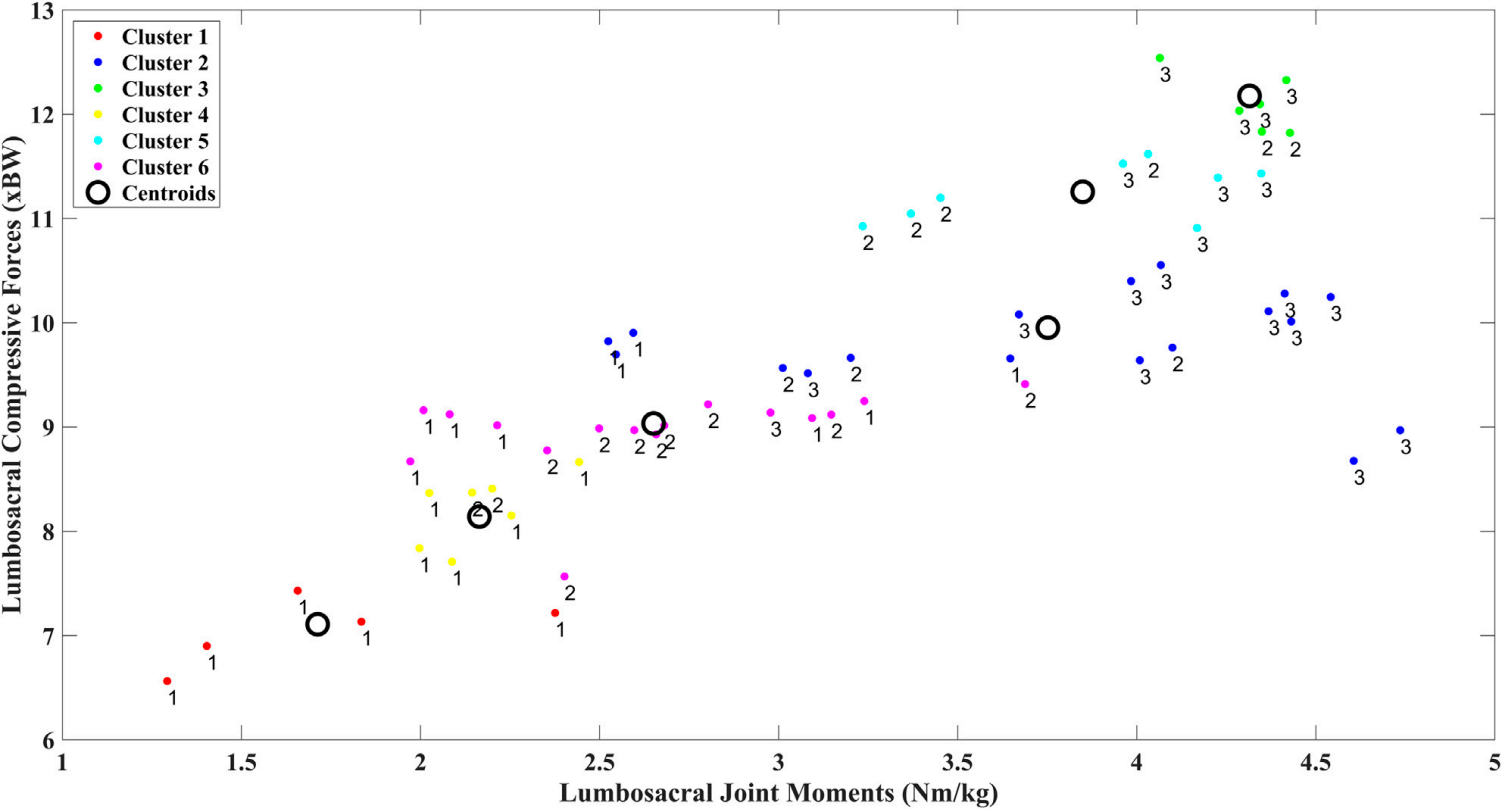
Clusters were identified using k-means to group body weight normalized peak compressive force against respective body weight normalized peak moments for the three risk levels.

**FIGURE 8:**
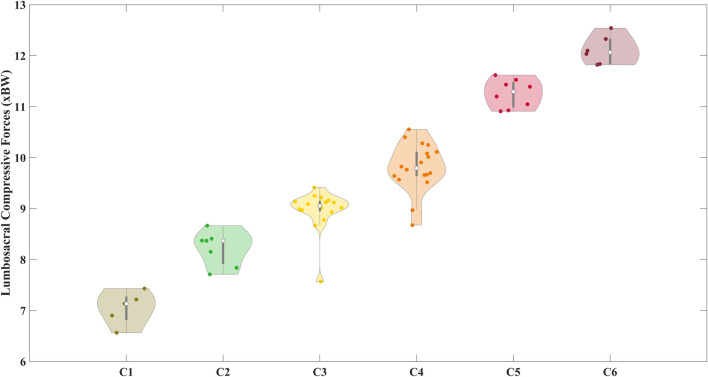
Distribution of the peak compressive forces within the clusters defined earlier. The clusters were significantly different from each other (p < 0.01).

Six clusters of compressive forces grouped with sagittal lumbosacral moments were identified (Figure 7). The compressive forces in these clusters were significantly different from each other (Table 2, all p < 0.01). The distribution of the peak compressive forces grouped in these clusters is shown in Figure 8. In Table 3, we see that the NIOSH risk levels are rather spread across these clusters.

**TABLE 2:** Two-sample t-test applied between the different clusters.

Cluster a	Cluster B	tstat
1	2	−5.6
	3	−15.3
	4	−17.5
	5	−23.1
	6	−24.7
2	3	−8.9
	4	−13.7
	5	−22.1
	6	−25.3
3	4	−10.2
	5	−22.8
	6	−28.6
4	5	−10.3
	6	−15.4
5	6	−5.9

**TABLE 3:** Comparing the new clusters with the NIOSH-based risk levels.

Risk levels/Clusters ->	C1	C2	C3	C4	C5	C6
LI1	5	5	6	4	0	0
LI2	0	2	9	3	4	2
LI3	0	0	1	11	4	4

## Discussion

This study estimated biomechanical loads on the lumbosacral joint using a large-scale physiologically valid pEMS model during lifting at different risk levels prescribed by the RNLE. We found that the objective parameters such as lumbosacral joint moments, compressive and shear forces increased with increasing risk. The study offers an objective and personalized approach to assess low-back loading which has potential in assessing individualized risk of injury at the workplace. Empirical tools for assessing loading and risk have been linked to low back pain and injury. Setting up longitudinal studies that monitor the probability of injury with the biomechanical parameters estimated by pEMS is needed to establish a direct link between them. This approach can be included in the toolbox of health and safety managers, and ergonomists to assess injury risk at the workplace.

The pEMS model was calibrated for each individual which results in a personalized estimate of their lumbosacral moments, compressive and shear forces. This accounts for their body weight, dynamic range of movement, as well as muscle activity during the task. The calibration identifies an optimal parameter musculoskeletal set that minimizes the joint moments estimated from the EMG-driven model with that of the inverse dynamics approach (Pizzolato et al., 2015). The data collected during the low and high risk lifting trials were used, allowing the model to generalize across lifting conditions. Thus, the pEMS provides a personalized, non-linear mapping of muscle activity and movement to joint moments, compressive, and shear forces.

Although the median peak EMG increased across the three risk conditions (Figure 5), only the Iliocostalis Lumborum muscle activity was significantly different across all the three risk conditions, while for the Longissimus Lumborum statistical differences were shown only between low and medium, and low and high-risk levels. Overall, with increasing risk, the user must increase the activation of their muscles to lift the object from a lower height. The muscle activity of the abdomen muscles was low across participants and showed no significant differences between the three risk conditions. We see clear differences in the peak lumbosacral joint moments in the sagittal plane across the risk levels (Figure 6A). The same can be seen for the distributions of peak compressive and shear (Figures 6B, C) forces. Therefore, the differences in risk levels suggested by RNLE are reflected in the biomechanical parameters estimated by data-driven person-specific musculoskeletal models, in line with our hypothesis.

However, there are two points of interest from these analyses. First, the joint forces estimated in the study were well above the limit of 3.4 kN proposed by NIOSH (Waters et al., 1993). The compressive forces acting on the lumbosacral joint estimated by the pEMS model include the passive and active forces generated by the trunk muscles. The median compressive forces ranged between 6.7 kN and 8.1 kN (Figure 6B) for an 80 kg person across the three risk levels. Furthermore, the shear forces were also quite high, measuring 1.7 kN and 4.1 kN for the low and high-risk conditions respectively (Figure 6C). Therefore, it is useful to consider whether the NIOSH limit underestimates the higher peak compressive forces during dynamic tasks.

Secondly, we see that the lumbosacral joint moments and compressive forces (Figure 6B) were bimodally distributed for the low high, and medium-risk levels respectively. This indicates that the risk levels denoted by RNLE do not encompass distinctly different risk levels at the biomechanical level (Zhivomirov, 2022). This was confirmed by our cluster analysis (Figures 7, 8). We found that using compressive forces, we can further demarcate the three risk levels into six distinct groups with unimodal distributions. Moreover, these groups were significantly different from each other (Table 2). Table 3 further shows that the risk levels do not necessarily fall into fixed categorical levels of low, medium, and high risk, and reflect a spread across these six groups. For instance, cluster 4 contains compressive forces that are assessed by the RNLE to be across all three risk levels. The cluster analysis also shows that the compressive forces are not well differentiated between the medium and high-risk levels. This suggests that the RNLE misses aspects of dynamic movements, which are rather captured by personalised EMG-driven approaches.

Although data were collected from a larger number of participants, due to technical issues, the study included a smaller subset of 7 individuals. This may limit the generalizability and the robustness of the study’s results. For this reason, a larger and more diverse dataset (inclusive of gender and race) needs to be collected and analysed to confirm the results obtained in this study. Indeed, gender diversity is necessary as men and women have different physiological risks of developing WLBDs due to differences in pain perception, fatigability, tendon properties, anthropometry, and muscular entities (Johansen et al., 2013; Sullivan et al., 2009).

## Limitations and future work

The current setup for pEMS is not applicable for the workplace. Requesting the health and safety managers to implement this process would be cumbersome. The ease of implementing the additional sensing for muscle activity and joint kinematics, and the wearability of the sensors must be addressed before the modelling approach can be implemented. Joint kinematics were estimated using a motion analysis system in this study. However, this can be replaced with either miniature IMUs placed on the body (García-de-Villa et al., 2023), or using a markerless approach (Lam et al., 2023). These techniques can remove the need for marker placement and calibration in the factory floor. Muscle activity was measured using EMG, which require proper sensor placement and efficient processing of the noisy signal. These signals were filtered using standard processing techniques (Winter, 2009) to account for the uncertainties. Additionally, due to a limited number of EMGs measured during the experimentation, we made some assumptions to match the EMGs collected to the different MTUs used in the model (Moya-Esteban et al., 2022). However, the wearability of these sensors can be improved using synergy-based approaches or clustering techniques. Using a synergy-based approach can reduce the number of EMG sensors required for this study to just two sensors (Rook et al., 2024). Alternatively, a garment with an array of sensors can be worn by the user, and clustering techniques (Simonetti et al., 2022) can be used to automatically identify the muscles of interest. Wearable sensorized garments integrated with IMUs and EMGs can thus offer an unobtrusive option to monitor the musculoskeletal health of the user in the workplace. This information can help track the impact of the workplace over time leading to better insights on the possibility of individualized risk at the workplace.

The musculoskeletal model used in this study is quite complex with 238 MTUs (Beaucage-Gauvreau et al., 2019). Calibrating the MTU parameters requires a few hours and is dependent on the computing power. Nonetheless, once a calibrated model has been generated per participant, this is applicable across several repetitions of the task being performed. The compressive force estimation performed in this study could be recommended for new employees. This represents a new option with a real-time assessment of person-specific compressive forces under real working conditions that can be an alternative to the methods already used for biomechanical risk assessment (e.g., the NIOSH protocol) (Moya-Esteban et al., 2023). Moreover, the LFB model used in the study did not include passive structures such as ligaments and tendons that play an important role in stabilizing the trunk during lifting (Meszaros-Beller et al., 2023). The joint moments and forces estimated in this study should be revisited with an improved model of the trunk at the cost of computational time.

Thus, using a person-specific EMG-driven musculoskeletal model, we could better demarcate the risk during the lifting activity objectively as the approach considers both muscle activity and the user’s pose. Based on the results of this study, and earlier studies (Dieën and Kingma, 2007; Faber et al., 2009; Kim and Zhang, 2017), we suggest future studies with larger and diverse populations to evaluate the ergonomic limits for lifting at the workplace. Moreover, longitudinal studies that relate the compressive and shear forces measured individually with the actual injuries that occur must be performed. These objective and personalized measures could inform the Standards for Human Ergonomics for lifting and carrying (ISO, 2003). Moreover, the revision of ergonomic limits must account for the progress in the development of several novel algorithms and indices, and wearable measurement solutions (Carmen et al., 2022; Chini et al., 2022; Le et al., 2017; Peppoloni et al., 2016; Ranavolo et al., 2015; Ranavolo et al., 2017; Ranavolo et al., 2018a; Ranavolo et al., 2018b; Varrecchia et al., 2018a; Varrecchia et al., 2021; Varrecchia et al., 2018b).

## Data Availability

The datasets presented in this study can be found in online repositories. The names of the repository/repositories and accession number(s) can be found below: https://humandatacorpus.org/. In detail, the data of this article are “INAIL Lifting Dataset 1” in the following session of repository: Lifting and Carrying – ISO 11228 – Human Data Corpus (https://humandatacorpus.org/lifting-and-carrying-iso-11228/).
